# Role of Venture Capital in Enterprise Innovation Under Psychological Capital and Heterogeneity of Entrepreneur Capital

**DOI:** 10.3389/fpsyg.2020.01704

**Published:** 2020-07-10

**Authors:** Chenjing Zhang, Di Mao, Mancang Wang

**Affiliations:** ^1^School of Economics and Management, Northwest University, Xi’an, China; ^2^Business School, University of Essex, Colchester, United Kingdom

**Keywords:** psychological capital, capital heterogeneity, venture capital, enterprise innovation, entrepreneur capital

## Abstract

This paper is to explore the impact of entrepreneurial psychological capital and capital heterogeneity on venture capital behavior, and further analyze the effect of venture capital on the innovation activities of enterprises. Based on the existing research results, this paper proposed hypotheses on the relationship between venture capital and technological innovation. According to the data samples of growth enterprises market (GEM) listed companies from 2010 to 2016, the main research variables were defined and a theoretical analysis model was constructed. The theory and empirical research clarify the relationship between venture capital and technological innovation. (1) According to the regression results of venture capital participation as well as innovation input and innovation output, the regression coefficients of venture capital participation are 0.609 and 0.203, which are significant at the levels of 10 and 1%, respectively. It indicates that venture capital participation has a positive impact on the innovation input and output of enterprises. (2) The coefficient of venture capital participation is positive, and the coefficient of $\bar{\mathrm{HHI}\times \mathrm{VC}}$ is significantly negative. Therefore, the degree of product market competition has a significant moderating effect on the relationship between venture capital participation and technological innovation. Venture capital provides funding support for technological innovation in startups. At the same time, because it holds a certain percentage of shares, it participates in enterprise innovation activities and provides guidance for companies to maintain profitable growth, thereby improving their innovation awareness and level. This research makes up for the shortcomings of the previous research model that uses a single dimension to measure technological innovation. As a result, this study comprehensively investigates the impact of venture capital on the innovation input and output of enterprises, enhancing the integrity and reliability of previous research conclusions.

## Introduction

In recent years, with the rapid development of China’s economy, enterprise innovation driven by innovative entrepreneurial activities has played an important role in economic and social development (Jiang et al., 2019). In the era of innovation as the main theme, innovation is a key factor for an enterprise to maintain its competitive advantage and promote long-term development. The survey found that more than 85% of a country’s economic growth comes from technological innovation. It is thus evident that technological innovation is essential to promote economic growth (Newth, 2016). As the foundation of China’s national economy, the manufacturing industry is the key supporting force to promote scientific and technological progress, and it is also the main direction for technological innovation (Sun et al., 2016). To strengthen the strategic plan for enterprise innovation, it is necessary to firmly put innovation at the core of the overall development, apply more innovative technologies, maximize economic benefits, and improve the market competitiveness of enterprises (Florida and Mellander, 2016).

Aiming at the funding limitations of startups in their innovation activities, venture capital can provide financial support to enterprises, thereby alleviating their financing problems. Through the summary of the related research on the relationship between venture capital and technological innovation in recent years, it is concluded there is a positive correlation. Venture capital can play a positive role in improving regional innovation performance by controlling resource flow and selection bias (Gu et al., 2018). For enterprise innovation, there is usually a significant breakthrough before the acquisition of venture capital investment. After the venture investment behavior, the level of enterprise innovation will also decline (Pierrakis and Saridakis, 2019). In the specific decision-making and implementation process of the enterprise’s innovative behavior, entrepreneurs, as decision-makers in business management, have the final decision-making power in the enterprise. Therefore, he/she has a decisive role in the control of innovative production and management. The psychological state is an important hidden characteristic of entrepreneurs. Their achievement motivation, risk-taking tendency, and creative spirit will affect the entire innovation process of the enterprise (Hua et al., 2016). “Positive psychological capital” takes the emphasis on the positive psychological power of people as the core. Entrepreneurs’ psychological capital includes positive psychological abilities, such as self-efficacy, optimism, and persistence. It enables enterprise employees to have confidence in the entrepreneurial team, adjust their mentality in a timely manner when facing difficulties, and ultimately succeed.

This paper is to explore the impact of entrepreneurial psychological capital and capital heterogeneity on venture capital behavior, and further, analyze the effect of venture capital on the innovation activities of enterprises. Based on the data samples of growth enterprises market (GEM) listed companies from 2010 to 2016, the relevant data for venture capital were collected, and the main research variables were defined, and a theoretical analysis model was constructed. The theory and empirical research clarify the relationship between venture capital and technological innovation. Through comprehensive examination over the impact of venture capital on the innovation input and innovation output of enterprises, it is confirmed that venture capital has a significant promotion effect on innovation input and output of enterprises.

## Literature Review

In the research of the relationship between venture capital and technological innovation, some scholars believe that venture capitalists not only provide capital support to affect the value of investment enterprises but also provide additional resources and support services. For example, marketing, human resource management, as well as research and development (R&D) management help enterprises add value. At the same time, these value-added mechanisms are realized by actively participating in the management of the company. It is conducive to reducing the moral hazard of management and increasing the degree of its R&D innovation efforts (Salomon, 2016). Venture capital enterprises generally invest in startups, and startups generally have relatively high risks. Therefore, venture capital enterprises are more inclined to urge the invested enterprises to carry out innovative activities to achieve excess returns to compensate for the high risks they bear (Wang et al., 2019).

According to Bottazzi et al. (2012), venture capital enterprises generally invest in startups, and startups generally have relatively high risks. Therefore, venture capital enterprises are more inclined to urge invested enterprises to carry out innovative activities to realize excess returns, compensating for the high risks they bear. In other words, venture capital has a greater expected return on the R&D investment of the enterprises it supports, which will encourage investment enterprises to invest more in R&D and innovation. In the research on the correlation between venture capital and technological innovation of enterprises, Tan et al. (2013) believed that for small and medium-sized enterprises in China, venture capital enterprises do not help the invested enterprises to add value during the IPO process, nor help them to improve innovation efficiency. It indicates that venture capital is not positively related to enterprise technological innovation.

Venture capital has developed in China for merely a short period of time, with little published data. Existing research does not comprehensively measure risk investment and technological innovation. Most studies only consider whether there is venture capital participation, but not the participation intensity of venture capital. For the measurement of technological innovation, only the intensity of scientific research innovation input or the number of patent applications is considered. Based on this, whether there is venture investment participation is considered, and the intensity of participation is measured by the proportion of venture capital holdings for a more in-depth analysis.

## Materials and Methods

### Entrepreneurial Psychological Capital

In enterprise practice, investigations have found that employees’ positive psychological resources will have a positive effect on the organization’s competitive advantage. From a psychological perspective, the theoretical basis of psychological capital is derived from the theory of positive organizational behavior. The concept of psychological capital originally refers to a series of life perspectives such as people’s perception of themselves and attitudes toward work (Bhatt and Ahmad, 2017). From the perspective of the trait theory school, psychological capital is an inherent trait of the individual, which affects the behavior and output of the individual. Also, it is the result of the combined action of innate and acquired. From the perspective of state school, psychological capital is a positive mental state. Psychological capital is an individual’s positive psychological ability. It is a positive state in which individuals treat work tasks, performance, and ultimately success in specific situations. It has a significant impact on individuals’ cognitive processes and work performance. The comprehensive theory holds that psychological capital includes both traits and state-trait mental qualities. It is a theory that combines state theory and trait theory.

Psychological capital can be obtained, maintained, and promoted autonomously in a variety of ways, both for enterprises and individuals. Individuals with good psychological capital are usually able to withstand challenges and become successful employees, managers, and entrepreneurs (Bailey et al., 2018). Confident, optimistic, and tenacious people are the most innovative and motivated. They can maximize their knowledge and skills to meet local conditions and achieve their life value while helping enterprises to continuously improve performance and profits. The psychological capital status of the internal group of the enterprise exceeds the human capital and social capital of the enterprise. It is a key element for the enterprise to maintain its competitive advantage. Although the psychological capital theory is more suitable for the fields of human resource management and active organizational behavior, the application of psychological capital in the field of technological innovation research also provides a good opportunity for it. The main reason is that under the effect of psychological capital, the employees of the enterprise continue to create value, develop their potential, and improve the performance of technological innovation.

The scholar Gao Na derived the seven-factor model of entrepreneurial psychological capital from the theoretical and empirical perspectives, as shown in Table 1 (Gao and Jiang, 2013). Among them, active procedures include calmly dealing with difficulties and overcoming obstacles as planned. It is consistent with toughness in connotation. Psychological capital focuses on the positive orientation of individual development. The human capital is oriented toward problem solving, focusing on the problems of enterprises and individuals. Psychological capital has become the core psychological element beyond human capital and social capital. Psychological capital can effectively be measured and managed. The performance is improved through investment and development, enabling organizations to gain a competitive advantage.

**TABLE 1:** Concrete contents of the seven-factor model of psychological capital.

Factor	Concrete content
Positive growth	It refers to the way of looking at life, the choice of goals, the consciousness of implementation, and the ability of action
Active coping	It refers to calmly dealing with difficulties, starting from the beginning to the end, overcoming difficulties, and continuously updating the knowledge structure
Enthusiastic innovation	It refers to the life full of passion and energy, dare to dare to do, live a challenging life, feel their own vitality
Keen and outstanding	It refers to critical thinking, keen market recognition, studious, and the pursuit of excellence
Self-efficacy	It refers to multi-task processing ability, ability to cope with challenges freely, self-belief, and efficacy expectation
Social intelligence	It refers to be aware of the motives and emotions of others and yourself, and have strong communication ability
Optimistic hope	It refers to an optimistic and peaceful state of mind and the process of realizing the goal requires willpower

### The Impact of Entrepreneur Capital Heterogeneity on Enterprise Innovation

**FIGURE 1:**
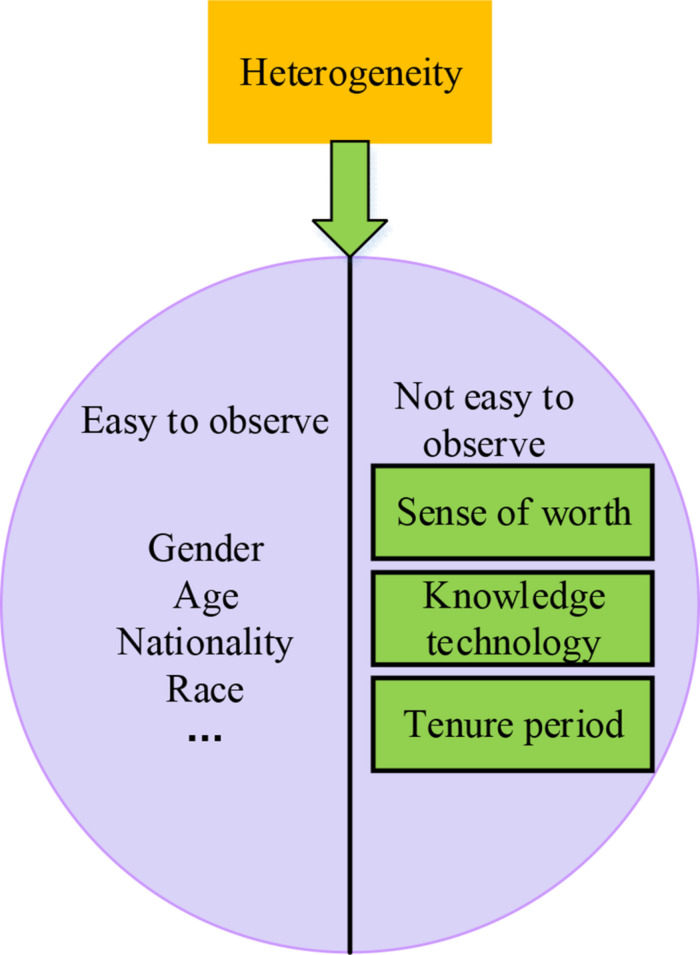
Heterogeneity classification results.

Heterogeneity refers to the uniqueness and difference of individuals or organizations from other subjects, contrary to the content of homogeneity. Heterogeneity can be divided into two aspects: easy to observe heterogeneity and not easy to observe heterogeneity. Among them, the content easy to observe includes age, gender, nationality, and race. The content not easy to observe includes a sense of worth and attitudes, knowledge, and technology accumulation, as well as the tenure period, as shown in Figure 1.

Combining research on the higher-order theory and human capital theory, entrepreneurial heterogeneity can be classified from three aspects: psychological behavior characteristics, demographic characteristics, and human capital characteristics (Muchira et al., 2019). Demographic characteristics reflect the heterogeneity of individuals to a certain extent, and there are obvious advantages. Entrepreneur demographics is an important variable that affects business performance. The higher-order theory provides a theoretical basis for studying the relationship between entrepreneur demographics and enterprise performance. To deeply analyze the cognitive foundation and values of entrepreneurs, they can be replaced by relevant indexes such as gender, age, nationality, and race in demography. Explicit demographic characteristics are important features of entrepreneurial heterogeneity, and recessive psychological behavior is also the key to determining entrepreneurial heterogeneity. Entrepreneurs’ risk appetite, personality traits, and other psychological characteristics variables can affect their judgments and behavioral tendencies, which in turn has an impact on enterprise profitability (Yao et al., 2019). Comprehensive research on psychological characteristics found that most of the literature emphasized the importance of entrepreneurial psychological behavior characteristics to enterprise development (Jing et al., 2017). They generally believe that achievement motivation, risk-taking tendency, tenacity, and innovative spirit are all typical characteristics of successful entrepreneurs in terms of psychological behavior characteristics. In addition, human capital, as the most important resource in total factor production, includes two aspects from the basic composition: knowledge ability and social capital. Entrepreneurs have high-level human capital, and the quality of human capital will have a direct impact on the production performance and innovation capabilities of enterprises.

Entrepreneur heterogeneity is a rich concept. Therefore, related research needs to specify a certain boundary range. At present, the gender and age of entrepreneurs are mainly used as demographic characteristics. The educational background and tenure period of entrepreneurs are used as human capital characteristics. These four variables were used as characteristic indexes of entrepreneur heterogeneity. Related research on enterprise performance and strategic decisions was conducted. Leaders with different education levels and tenure periods would directly affect the innovation activities and performance of enterprises (Cetindamar and Ozkazanc, 2017). The higher the education level of entrepreneurs, the more obvious the improvement of enterprise performance. Especially, when the entrepreneurs have been in the enterprise for a long time, the positive impact of educational level on enterprise performance is more obvious. In terms of strategic decisions, there is a positive correlation between the education level of entrepreneurs and the enterprise’s internationalization strategy. In other words, the richer the diversified career background of the entrepreneur, the more they tend to diversify the strategy, and the better the results of the chosen strategic decisions for the enterprise. The longer an entrepreneur works, the more comprehensive the issues considered. Therefore, more attention is paid to the balance of the interests of all parties, and the slower the speed of corresponding strategic decisions.

### The Relationship Between Venture Capital and Enterprise Technology Innovation

In the real economic environment, there are obvious drawbacks for reliance on the theory of rational expected utility. Generally, it is difficult to explain the investment decision behavior in the real market economy, and the rational perspective has serious perceived risks for the uncertainty of decision results. Thus, the impact of human behavior on economic decisions began to receive attention. Competition in the economic and social markets does not exist in a static state, and the existence of market economic risks is a normal state. Venture capital is an entrepreneurship capital, mainly aimed at startups, providing them with financial support and obtaining shares in the company. Venture capital originated from the American Research and Development Corporation (ARD), which invested $ 70,000 in the Digital Equipment Company (DEC) in the 1940s, holding a 7% stake. Ten years later, ARD’s shareholding was worth the US $ 355 million. Thus, it became a classic case of venture capital (Migendt et al., 2017; Pradhan et al., 2018; Kim, 2019). Venture capital is to invest funds in some high-tech projects with relatively high potential risks and high technological innovation capabilities to help market high-tech achievements. Investors are to get high returns from the realization of new technology commercialization.

Some companies with potential value-added space will face many difficulties when they startup. The lack of funds is the most critical issue that has the greatest impact on the development of enterprises. Generally, companies with potential are almost high-tech industries. In the process of development, they not only face the uncertain high risks in the process of technological innovation, but also face the risks brought by the incomplete management of enterprises, including technology, capital, management, and market. Therefore, for investment, there are huge returns brought by high-tech. Once the investment is successful, the company’s technological competitive advantage will quickly create market monopoly benefits. At the same time, there is a series of uncertain high loss risks in the startup process. The main reason is that comparing the high-tech with traditional industry technologies, the market is not mature and stable (Vorontsova and Vorontsov, 2015). For enterprises with innovative value but lack of financial support, venture capital experts can explore the value of the enterprise through research, judgment, and investment decisions. Venture capital activities need to be completed by two entities, namely, professional investment talents and capital holders. The capital is injected by investors and capital management is carried out by venture capital experts. Professional investment talents have a high degree of acuity and insight when evaluating the development of scientific and technological innovation products. They can reasonably invest capital in high-tech industries with high value-added potential, thereby providing conditions for the development and growth of these enterprises’ technological innovation. While the high premium brought by technological innovation is realized, it has also created richer returns for venture capital providers.

The high-tech enterprises have not yet been recognized by the market for their technologies and products. Also, the company’s overall operating capabilities are relatively immature. Thus, it is difficult for external investors to provide financial support for their technological innovation in the face of numerous market uncertainties. Some scholars have conducted empirical investigations and found that compared with enterprises without venture capital participation, enterprises with venture capital participation have stronger innovation capabilities and more patents (Pradhan et al., 2017). The main reason is that venture capital can provide a fund guarantee for enterprise R&D, thereby promoting investment objects to focus more on innovation output. But at the same time, there is also a point of view that the purpose of some venture capital enterprises is to seek benefits rather than technological innovation (Majid and Agassi, 2017). Therefore, if investors find that there are other better ways to make profits, they often make the investment object change the operating entity and ignore technological innovation. Therefore, venture capital does not have a positive effect on technological innovation in some cases. Studies have shown that enterprises have more innovation activities before venture capital participation. Instead, after the venture capital participation, the original technology innovation focus has been transferred to other activities with higher returns. Venture capital does not promote innovation and transfers the focus of the enterprise, which has a negative impact on the enterprise’s willingness to innovate and the development of innovation activities.

### Research Hypothesis

Synthesizing the existing research results, Western scholars mainly proceed from the perspective of mature capital markets. They believe that cross-border investment has many uncertainties because it increases the cost of distance and has to deal with the problems caused by the institutional and market environments of different countries. However, the overseas investment can broaden the scope of potential investments and tap into more startups with potential value (De et al., 2017). For the technological innovation of enterprises only, foreign-invested venture capital has certain advantages. Because investors are concerned about cultural differences, more comprehensive enterprise and market surveys are conducted, as well as operations in the selection of investment projects will be more standardized.

Due to the long R&D cycle of high technology, large capital consumption, and uncertainty before the technology maturity, it is difficult for enterprises to obtain loans from traditional financial institutions for their technological innovation activities. Venture capital, as a new type of financing method, provides fund support for enterprises’ technological innovation. It helps new technologies with market development prospects be transformed into actual productivity and promotes the development of innovative activities while obtaining market value. Venture capital can help enterprises with inadequate management systems to provide non-capital consulting management services such as R&D, and operation management while solving enterprise financing problems. The investment enterprise formulates a series of operational development strategies for it by combining its management experience with the current status of the investment object. In addition, investors will pay attention to the improvement of the enterprise innovation ability, and use their professional market prior knowledge to help the enterprise form contacts with external units such as the government, customers, and suppliers. Then, it can minimize the degree of information asymmetry between the enterprise and other institutions. Information asymmetry in the technological innovation environment may cause the innovation results to be easily imitated. Thus, the innovation process requires more fund and energy support. Venture capital can participate in enterprise governance in the form of equity investment, and comprehensively supervise the enterprise, thereby promoting the technological innovation of the enterprise. In summary, the following hypothesis is proposed for the relationship between venture capital and technological innovation.

H1: Venture capital will have a positive impact on the technological innovation of enterprises.

In China’s market economy system, product market competition is an important external governance mechanism, which is greatly significant for improving the operating efficiency of enterprises, thereby affecting their technological innovation. Current research confirms that product market competition can alleviate the problem of information asymmetry and bring the interests of managers and shareholders closer to each other. In this case, investors can more effectively supervise the operators of the enterprise and avoid situations in which the operators seek private profits. The fierce competition in the product market will put a certain amount of pressure on the company’s internal governance, which will prompt enterprises to strengthen the positive impact of internal control on innovation input (Wu et al., 2019). To maintain the long-term stable benefits of enterprises, venture capitalists will guide enterprise managers to more efficiently formulate innovative strategic plans, thereby promoting the development of technological innovation in enterprises. Therefore, this paper makes the following hypothesis about the impact of product market competition on the relationship between venture capital and enterprise technology innovation.

H2: The higher the degree of competition in the product market, the greater the impact of venture capital on enterprise technological innovation.

Based on data collected on venture capital participation and the proportion of venture capital holdings, the main research variables were defined. The theoretical analysis model was constructed according to research goals. The STATA14.0 version of data statistics software was used for descriptive statistical analysis, correlation test, and regression analysis. Finally, the obtained results tested the proposed hypothesis, and the data results were interpreted and analyzed.

### Sample Data and Variable Design

Due to the rapid growth of GEM companies, many enterprises have not withdrawn their venture capital after listing, which is more in line with the research content. Also, the R&D related data of the GEM companies are more complete. Therefore, the data of GEM listed companies from 2010 to 2016 were taken as the sample in this study. The listed companies with missing related data, ST enterprises, and financial listed companies were excluded. Finally, a total of 2535 sample observations were determined. This study used the CSMAR Guotai’an database for data acquisition. All continuous variables were subjected to 1% winsorize to avoid the impact of extreme values on the accuracy of the research results. Data collection and statistical analysis were performed on the original data samples using EXCEL 2016 and STATA14.0 software.

(1) Explained variable: For the measurement of technological innovation, this paper chose two variables of R&D investment intensity and patent application number for evaluation. The measurement of innovation input was based on the proportion of R&D investment in operating income. Regarding the measurement of innovation output, it was more reasonable to use the index of the number of patents to evaluate innovation output at the enterprise level.
(2) Explanatory variable: (i) Venture capital participation: It was a dumb variable. Those with venture capital participation were 1 and those without venture capital participation were 0. (ii) Shareholding ratio of venture capital: Venture capital was an equity investment, and the shareholding ratio could reflect the participation level of venture capital.
(3) Moderating variable: The degree of competition in the product market was evaluated by the Herfindahl index (HHI). HHI was calculated based on the operating income of listed companies. Where *n* represents the number of enterprises in the industry where enterprise *i* belongs. *X*_*i*_ represents the annual business income of each individual enterprise in the industry. *X* represents the sum of the annual business income of all enterprises in the industry. The calculation equation of HHI is expressed as shown below.

(1) $$\text{HHI}=\Sigma_{1}^{n}{\left(X_{i}/X\right)}^{2}$$

(4) Control variables: Enterprise scale, enterprise life span, profitability, solvency, and year.

The specific definitions of various variables in this study are shown in Table 2.

**TABLE 2:** Specific definitions of various variables.

Variable type	Variable name	Symbol	Definition
Explained variable	Innovation input	RDI	The proportion of R&D investment in operating revenue innovation output
	Innovation output	PT	Total number of patent applications
Explanatory variable	Venture capital participation	VCdum	Venture capital participation is 1, otherwise 0
	The shareholding ratio of venture capital	VCshare	The total shareholding ratio of venture capital among the top 10 shareholders
Moderating variable	Product market competition	HHI	Herfindahl index
Control variable	Enterprise scale	Size	Year—year of establishment
	Enterprise life span	Age	Net profit/average balance of total assets
	Profitability	Roa	Total liabilities/total assets
	Solvency	Lev	The logarithm of total assets
	Year	Year	Set 2010 as the base period, with a time span of 7 years and six annual virtual variables

### Model Building

In this study, the impact of venture capital on technological innovation of the enterprise was explored to verify H1. If the sign of α_1_ is significantly positive, it means that venture capital can promote the innovation input or output of the enterprise. The equations for building the model are expressed as shown below.

(2) $$\begin{array}{c} \mathrm{RDI}=\alpha_{0}+\alpha_{1}\mathrm{VC}+\alpha_{2}\mathrm{Age}+\alpha_{3}\mathrm{Roa}+\alpha_{4}\mathrm{Lev}+\alpha_{5}\\ \mathrm{Size}+\Sigma \mathrm{Year}+\varepsilon \end{array}$$

(3) $$\begin{array}{c} \mathrm{PT}=\alpha_{0}+\alpha_{1}\mathrm{VC}+\alpha_{2}\mathrm{Age}+\alpha_{3}\mathrm{Roa}+\alpha_{4}\text{Lev}+\\ \alpha_{5}\mathrm{Size}+\Sigma \mathrm{Year}+\varepsilon \end{array}$$

In this study, the impact of product market competition on the relationship between venture capital and technological innovation of the enterprise was explored to verify H2. If the sign of α_3_ is negative, it means that as the degree of market competition decreases, venture capital is gradually weakening the promotion of technological innovation of the enterprise. Models (4) and (5) are constructed based on Models (2) and (3).

(4) $$\begin{array}{c} \mathrm{RDI}=\alpha_{0}+\alpha_{1}\bar{\text{VC}}+\alpha_{2}\bar{\text{HHI}}+\alpha_{3}\bar{\mathrm{HHI}\times \mathrm{VC}}+\alpha_{4}\mathrm{Age}+\\ \alpha_{5}\mathrm{Roa}+\alpha_{6}\mathrm{Lev}+\alpha_{7}\mathrm{Size}+\Sigma \mathrm{Year}+\varepsilon \end{array}$$

(5) $$\begin{array}{c} \mathrm{PT}=\alpha_{0}+\alpha_{1}\bar{\text{VC}}+\alpha_{2}\bar{\text{HHI}}+\alpha_{3}\bar{\mathrm{HHI}\times \mathrm{VC}}+\alpha_{4}\mathrm{Age}+\\ \alpha_{5}\mathrm{Roa}+\alpha_{6}\mathrm{Lev}+\alpha_{7}\mathrm{Size}+\Sigma \mathrm{Year}+\varepsilon \end{array}$$

## Results

### Descriptive Statistics

**FIGURE 2:**
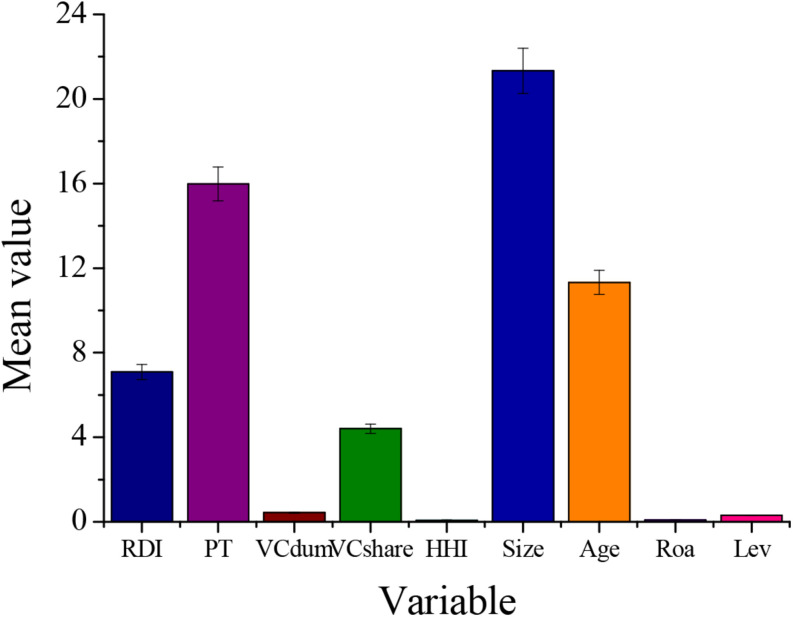
Descriptive statistics for each variable.

The descriptive statistics of each variable are shown in Table 3 and Figure 2. According to the statistical results, the maximum value of enterprise R&D investment intensity is 98.56%, and the maximum value of enterprise innovation output is 670. However, the minimum of these two explained variables is 0. This shows that there is still a large gap in technological innovation between GEM listed companies. The proportion of venture capital holdings is 4.403%, of which the highest proportion of venture capital holdings is as high as 80.2%, indicating that among GEM listed companies, venture capital occupies a certain proportion of ownership. The product market competition intensity is 7.5%. The maximum and minimum values are 90.7 and 1.7%, respectively. More than half of the enterprises are facing a more competitive market. Due to the existence of monopolies in the industry, the competitiveness of different enterprises’ products is quite different.

**TABLE 3:** Descriptive statistics of each variable.

Variable	*N*	Mean value	Standard deviation	Minimum	Maximum
RDI	2535	7.088	7.022	0	98.56
PT	2535	15.983	35.80	0	670
VCdum	2535	0.429	0.502	0	1
VCshare	2535	4.403	8.784	0	80.2
HHI	2535	0.075	0.078	0.017	0.907
Size	2535	21.328	0.746	17.598	25.411
Age	2535	11.325	4.431	1	29
Roa	2535	0.083	0.060	–0.472	0.568
Lev	2535	0.312	0.155	0.010	0.867

### Results of Correlation Analysis

The regression results of the correlation coefficients between the intensity of R&D investment, the number of patent applications, and the control variables are shown in Table 4. It can be seen that the intensity of R&D investment, the number of patent applications, and the proportion of venture capital holdings are significantly positively correlated at the 1% level. Enterprise innovation output and venture capital participation are significantly positively correlated at the 1% level, which initially confirmed the expected hypothesis proposed earlier. The correlation coefficients of the regression between the variables are within the acceptable range, which indicates that the model used does not have obvious multicollinear interference.

**TABLE 4:** Correlation coefficient regression results of main variables (*n* = 2535).

Variable	1	2	3	4	5	6	7	8
RDI	1							
PT	0.048	1						
VCdum	0.047	0.065*	1					
VCshare	0.073*	0.022	0.186*	1				
HHI	−0.083*	0	–0.020	0.006	1			
Size	−0.081*	0.230*	–0.064	0.011	0.012	1		
Age	−0.056*	−0.055*	−0.053*	–0.025	−0.054*	0.092*	1	
Roa	−0.272*	0.119*	–0.006	0.009	0.027*	0.146*	−0.228*	1

*Indicates a significant positive correlation at the 1% level.

### Analysis of the Regression Results of the Impact of Venture Capital on Technological Innovation of Enterprises

**FIGURE 3:**
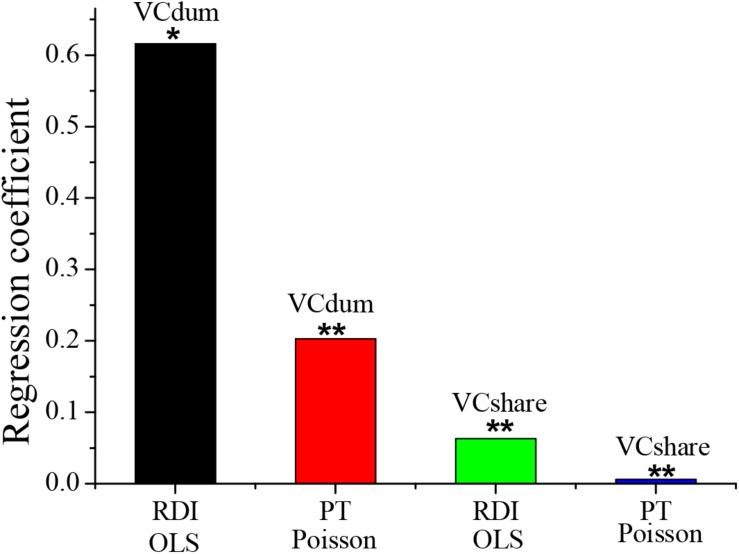
The impact of venture capital on technological innovation of enterprises. * and ** indicate significant positive correlations at 10 and 1% levels, respectively.

This paper took the innovation input and output of the enterprise as the dependent variables to test Model (1) and Model (2), thereby clarifying the impact of venture capital on technological innovation. The least-squares method was used to test the impact of innovation input, and the Poisson regression was used to test the impact of the number of patent applications. The test results are shown in Table 5 and Figure 3. According to the regression results of venture capital participation as well as innovation input and innovation output, the regression coefficients of venture capital participation are 0.609 and 0.203, which are significant at the levels of 10 and 1%, respectively. It indicates that venture capital participation has a significantly positive impact on the innovation input and output of enterprises. According to the regression results of the proportion of venture capital shareholding as well as innovation input and innovation output, the regression coefficient of the proportion of venture capital shareholding is significantly positive. It indicates that the higher the proportion of venture capital shareholding, the more beneficial the technological innovation of the enterprise. Thus, H1 is true.

**TABLE 5:** Impact of venture capital on technological innovation of enterprises.

Variable	RDI		PT	
	OLS	OLS	Poisson	Poisson
VCdum	0.616	−	0.203	−
VCshare	−	0.063	−	0.006
Size	–0.618	–0.635	0.586	0.593
Age	–0.064	–0.068	–0.033	–0.035
Roa	8.302	–8.342	2.110	2.153
Lev	–8.680	–9.048	0.883	0.880

In the process of the positive impact of venture capital on the technological innovation of enterprises, some other variables will play a moderating effect. This paper took product market competition as a moderating variable and builds Model (4) and Model (5) to explore the effect of this variable. The moderating effects of product market competition in the relationship between venture capital and technological innovation are shown in Table 6. The coefficient of venture capital participation is positive, and the coefficient of $\bar{\mathrm{HHI}\times \mathrm{VC}}$ is significantly negative. Therefore, the degree of product market competition has a significant moderating effect on the relationship between venture capital participation and technological innovation. With the decrease in the degree of competition in the product market, the positive correlation between the proportion of venture capital shareholding and technological innovation has weakened significantly. The process proves that H2 is true.

**TABLE 6:** Moderating effects of product market competition in the relationship between venture capital and technological innovation.

Variable	RDI		PT	
	OLS	OLS	Poisson	Poisson
VCdum	0.535*	−	0.192**	−
$\bar{\text{HHI}}\times \mathrm{VCdum}$	−13.972**	−	−3.003**	−
$\bar{\text{VCshare}}$	−	0.075**	−	0.003**
$\bar{\text{HHI}}\times \bar{\text{VCshare}}$	−	−0.621**	−	−0.068**
$\bar{\text{HHI}}$	−5.179**	−7.366**	–0.000	−0.618**

* and ** indicate significant positive correlations at 10 and 1% levels, respectively.

## Discussion

Entrepreneurs actively identify opportunities and resources contained in the environment. They discover, evaluate, and develop entrepreneurial opportunities, effectively improving entrepreneurial performance. Under the severe competitive pressure, entrepreneurs’ self-confidence and optimism can often help them to look at the environment and their abilities more positively. Also, they set higher goals for enterprises, and are more proactive in action, thereby improving enterprise performance. For entrepreneurs, venture capital is an investment method that shares risks and benefits. Therefore, there is an initiative in selecting investment objects, which is an investment decision based on comprehensive professional research and evaluation. In investment evaluation, generally relatively low-risk and relatively mature enterprises are selected, and certain guarantees are required to provide investors to recoup their investment (Li et al., 2019). Venture capital provides fund support for R&D at the stage of technology innovation input. Venture capital can hold a certain percentage of shares, participate in the supervision and governance of the enterprise, and effectively promote the continuous development of technological innovation of the enterprise (Obschonka et al., 2018; Chen, 2019; Yu et al., 2019).

This paper is to explore the impact of entrepreneurial psychological capital and capital heterogeneity on venture capital behavior, and further, analyze the effect of venture capital on the innovation activities of enterprises. Based on the research of venture capital and technological innovation of the enterprise, the impact of different factors on the relationship between the two is analyzed. In this paper, the data of GEM listed companies from 2010 to 2016 were selected as the sample for empirical analysis (Zheng and Liu, 2020). According to the regression results of venture capital participation as well as innovation input and innovation output, the regression coefficients of venture capital participation were 0.609 and 0.203, which were significant at the levels of 10 and 1%, respectively. Evidently, the results indicate that venture capital participation has a positive impact on the innovation input and output of enterprises. The coefficient of venture capital participation is positive, and the coefficient of $\bar{\mathrm{HHI}\times \mathrm{VC}}$ is significantly negative. Therefore, the degree of product market competition has a significant moderating effect on the relationship between venture capital participation and technological innovation. The above empirical results verify H1 and H2 proposed in this paper. Also, it is confirmed that venture capital has a positive effect on enterprise innovation.

## Conclusion

Venture capital provides funding support for technological innovation in startups. At the same time, because it holds a certain percentage of shares, it participates in enterprise innovation activities and provides guidance for companies to maintain profitable growth, thereby improving their innovation awareness and level. The higher the proportion of venture capital holdings, the more beneficial the technological innovation of the enterprise. With the decrease in the degree of competition in the product market, the positive correlation between the proportion of venture capital shareholding and technological innovation has weakened significantly.

The main contribution of this paper is that based on the “input–output” process of the innovation, it makes up for the shortcomings of the single-dimensional measurement of technological innovation in previous research models. The impact of venture capital on the innovation input and innovation output of enterprises is comprehensively investigated. It is found that venture capital has a significant role in promoting innovation input and output of enterprises. It enriches and deepens the existing empirical research as well as enhances the completeness and reliability of previous research conclusions. The research in this paper has some shortcomings, mainly due to the limitation of data availability. Therefore, the evaluation indexes are not comprehensive enough. In the following research, more moderating variables should be included to analyze the impact of venture capital on technological innovation.

## Data Availability Statement

The raw data supporting the conclusions of this article will be made available by the authors, without undue reservation.

## Ethics Statement

The studies involving human participants were reviewed and approved by the University of Essex Ethics Committee. The patients/participants provided their written informed consent to participate in this study.

## Author Contributions

CZ contributed to writing—original draft preparation. DM contributed to writing—review and editing. MW was responsible for visualization and supervision. All authors contributed to the article and approved the submitted version.

## Conflict of Interest

The authors declare that the research was conducted in the absence of any commercial or financial relationships that could be construed as a potential conflict of interest.
